# A Two-Gene Balance Regulates *Salmonella* Typhimurium Tolerance in the Nematode *Caenorhabditis elegans*

**DOI:** 10.1371/journal.pone.0016839

**Published:** 2011-03-02

**Authors:** Elizabeth K. Marsh, Maaike C. W. van den Berg, Robin C. May

**Affiliations:** 1 School of Biosciences, University of Birmingham, Birmingham, West Midlands, United Kingdom; 2 Laboratory of Physiological Chemistry, University Medical Center Utrecht, Utrecht, The Netherlands; University of Washington, United States of America

## Abstract

Lysozymes are antimicrobial enzymes that perform a critical role in resisting infection in a wide-range of eukaryotes. However, using the nematode *Caenorhabditis elegans* as a model host we now demonstrate that deletion of the protist type lysozyme LYS-7 renders animals susceptible to killing by the fatal fungal human pathogen *Cryptococcus neoformans,* but, remarkably, enhances tolerance to the enteric bacteria *Salmonella* Typhimurium. This trade-off in immunological susceptibility in *C. elegans* is further mediated by the reciprocal activity of *lys-7* and the tyrosine kinase *abl-1*. Together this implies a greater complexity in *C. elegans* innate immune function than previously thought.

## Introduction

The nematode *Caenorhabditis elegans* is now firmly established as a powerful model system for the study of host-pathogen interactions [1], [2], [3]. Although many aspects of innate immunity are shared with higher vertebrates [4], nematodes lack a cell-mediated immune system and thus rely upon secreted antimicrobial molecules for a systemic immune response to pathogenic challenge [2].

One such group of antimicrobial enzymes, lysozymes, are evolutionarily ancient proteins that are actively lytic against a range of microbes [5]. Whereas many organisms have only one or two lysozyme genes, *C. elegans* has a family of at least ten differentially regulated genes that are predicted to show significant functional diversity [6], [7]. Within the *C. elegans* lysozyme family, *lys-7* has been the most extensively studied. Expression of this molecule is strongly induced upon exposure of the animal to the pathogenic bacteria *Serratia marcescens* [8], *Microbacterium nematophilum* [9] and *Salmonella* Typhimurium [10]. Furthermore, *lys-7* knockout animals show enhanced susceptibility to *M. nematophilum,* indicating that *lys-7* has a protective function against this pathogen [9].

Here we examine the function of LYS-7 during *C. elegans* infection with a number of human pathogens. Surprisingly, we show that LYS-7 acts together with the tyrosine kinase ABL-1 to regulate an immunological balance in which resistance to *C. neoformans* comes at the cost of susceptibility to *S.* Typhimurium. Since this phenotype is independent of infectious burden or disease persistence, our data suggest a novel function of LYS-7 in regulating pathogen tolerance in *C. elegans*.

## Results

### *lys-7* mutant animals are hyper-susceptible to killing by *Cryptococcus neoformans*

**Figure 1:**
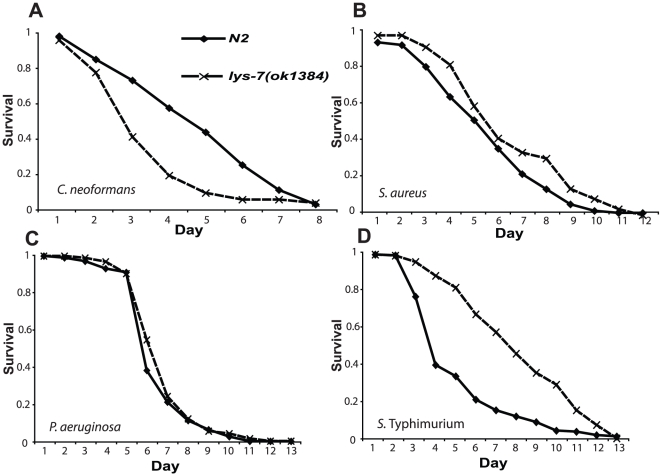
*lys-7* mutant nematodes are hypersensitive to cryptococcal infection, but resistant to *S*. Typhimurium. *lys-7* knockout animals show reduced survival relative to N2 upon infection with *C. neoformans* (A); p<0.0001, [*lys-7(ok1384)* n = 106; N2 n = 151], but wild type sensitivity to *S. aureus* (B) and *P. aeruginosa* (C), p>0.05, [*lys-7(ok1384)* n = 131; N2 n = 168] and p>0.2, [*lys-7(ok1384)* n = 104; N2 n = 111] respectively. Surprisingly, however, *lys-7* mutant animals exhibit enhanced resistance to *S*. Typhimurium (D); p<0.0001, [*lys-7(ok1384)* n = 191; N2 n = 242].

We, and others, have previously shown that the fatal fungal human pathogen *Cryptococcus neoformans* kills *C. elegans* [11], [12]. A preliminary gene expression study of two *C. elegans* strains, *daf-2(e1370)* and *fem-1(hc17)*IV, that are intrinsically resistant to killing by the fungus revealed that *lys-7* expression was strongly and constitutively induced in these animals (R. C. May, unpublished data). We therefore hypothesised that LYS-7 may play an important role in mediating resistance of the worm towards *C. neoformans.* In line with this prediction, a *lys-7* knockout strain (ok1384) showed wild type brood size and longevity under non-infectious conditions (Figs S1A and B; Methods S1), but severely reduced survival following exposure to the fungus (Fig 1A).

### *lys-7* mutant animals are resistant to killing by *Salmonella* Typhimurium

In order to establish whether the loss of LYS-7 influenced immunity to other pathogens, we exposed *lys-7* knockout animals to three organisms previously shown to be pathogenic towards *C. elegans*; the Gram-positive bacterium *Staphylococcus aureus* [13] and two Gram-negative bacteria *Pseudomonas aeruginosa* [14], [15], [16] and *Salmonella enterica* serovar Typhimurium [17], [18]. The susceptibility of *lys-7* knockout animals to *S. aureus* and *P. aeruginosa* was indistinguishable from that of the wild type strain (Figs 1B and C), suggesting that the loss of LYS-7 does not damage the worm immune response to all pathogens. In marked contrast, however, *lys-7* knockout animals are strongly resistant to killing by *S*. Typhimurium (Fig 1D). Identical effects were seen with a second, independent, allele: *lys-7(ok1385)* (Figs S1C and D), and an additional *S*. Typhimurium strain 14028 s (Fig S1E), indicating that this phenomenon, which we refer to as “balanced immunity”, is not allele or strain specific.

### Resistance to *S.* Typhimurium is mediated by genetic compensation

We considered that the loss of *lys-7* may trigger compensatory up-regulation of other genes that result in the observed resistance to *S.* Typhimurium. Expression analysis identified only four genes that are significantly up-regulated in both *lys-7* knockout strains (ok1384 and ok1385) in comparison to wild type animals: *abl-1* (2.65±0.94 fold up-regulation), *fat-5* (1.82±0.15 fold up-regulation), *clec-60* (1.59±0.08 fold up-regulation), and *rga-6* (1.92±0.089 fold up-regulation).

**Figure 2:**
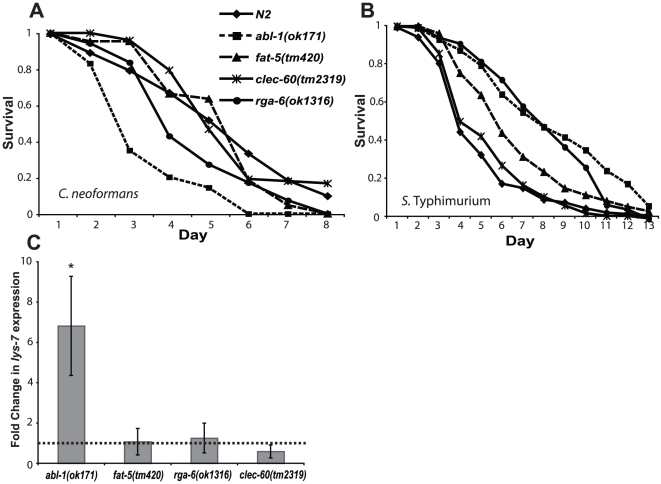
*abl-1* and *rga-6* mutant animals phenocopy *lys-7* susceptibility patterns. Knockout *C. elegans* strains for the four genes which were identified by a microarray to be up-regulated in the *lys-7* mutant background were assayed for survival upon infection with both *C. neoformans* and *S*. Typhimurium. The survival of *abl-1(ok171)* and *rga-6(ok1316)* animals was significantly lower than wild type animals upon *C. neoformans* infection (A), p<0.0001 and p<0.01 respectively. The remaining animals were unchanged from wild type survival, p>0.2, [N2 n = 186; *abl-1(ok171)* n = 130; *rga-6(ok1316)* n = 192; *fat-5(ok460)* n = 100; *clec-60(tm2319)* n = 110]. *abl-1(ok171)*, *rga-6(ok1316)* and *fat-5(tm420),* but not c*lec-60(tm2319)*, animals exhibited enhanced resistance to *S*. Typhimurium (B), p<0.0001 in each case, [N2 n = 170; *abl-1(ok171)* n = 222; *rga-6(ok1316)* n = 100; *fat-5(ok460)* n = 162; *clec-60(tm2319)* n = 125]. (C) *abl-1* mutant animals up-regulate *lys-7* expression (average induction of 6.8 fold; p<0.05), whereas *lys-7* expression is unchanged from wild type in the remaining mutant backgrounds. Data represent the mean expression of three independent experiments, ± S.E.M.

We obtained knockout strains for each of these genes and tested their susceptibility to killing by *C. neoformans* and *S.* Typhimurium (Figs 2A and B). Remarkably, animals lacking either *abl-1* or *rga-6* were hypersensitive to *C. neoformans* but resistant to *S.* Typhimurium infection; a survival pattern which phenocopies that of the *lys-7* knockout animals. *fat-5* knockout animals show slight resistance to *S.* Typhimurium but a normal susceptibility to *C. neoformans*, whilst *clec-60* mutant animals are indistinguishable from wild type animals in their survival following exposure to either pathogen.

We next used qRT-PCR to assess whether *lys-7* expression was altered in these knockout animals under normal culture conditions. *lys-7* expression remained unchanged in *fat-5, clec-60* and *rga-6* knockout animals, but, interestingly, was significantly up-regulated in *abl-1* mutants (Fig 2C). Thus *lys-7* and *abl-1* show reciprocal regulation of expression and identical loss-of-function phenotypes upon infectious challenge.

Importantly, the *abl-1* phenotype is not allele specific (Figs S2A and B) nor do *abl-1* animals show reduced lifespan, brood size or resistance to *S. aureus* infection (Figs S2C, D and E), indicating a specific role for ABL-1 in mediating immunological tolerance to *S.* Typhimurium.

### Reciprocal expression of *lys-7* and *abl-1* is required for tolerance to *S.* Typhimurium

**Figure 3:**
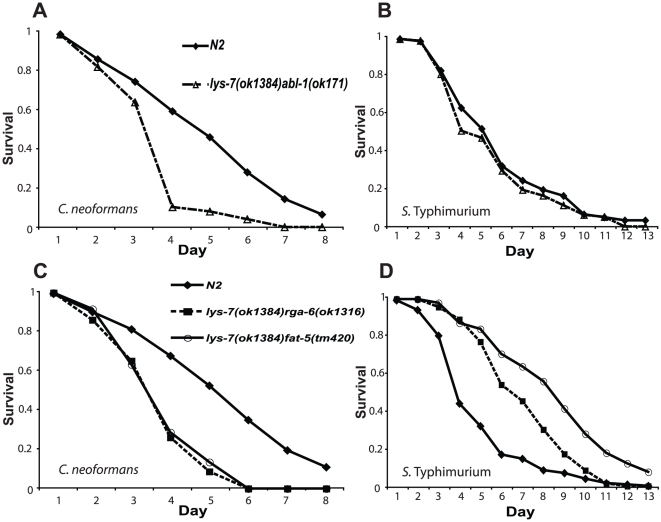
*lys-7;abl-1* double mutants suppress the tolerance resistance phenotype. *lys-7(ok1384)abl-1(ok171)* double mutants remain hypersensitive to *C. neoformans* (A), similar to their corresponding single mutants; p<0.0001, [*abl-1(ok171)lys-7(ok1384)* n = 133; N2 n = 150], but the resistance exhibited by the single mutants to *S*. Typhimurium infection (B) has been completely suppressed; p>0.2, [*abl-1(ok171)lys-7(ok1384)* n = 110; N2 n = 94]. Double mutants between *lys-7* and *fat-5* or *lys-7* and *rga-6 w*ere hypersensitive to *C. neoformans* infection (C), p<0.0001 in both cases, [N2 n = 207; *lys-7(ok1384)fat-5(tm420)* n = 100; *lys-7(ok1384)rga-6(ok1316)* n = 100]. Similarly, the resistance to *S*. Typhimurium exhibited by the *lys-7* knockout strain was either unaltered (*fat-5*) or only slightly reduced (*rga-6*) by these secondary mutations (D), [N2 n = 170; *lys-7(ok1384)fat-5(tm420)* n = 100; *lys-7(ok1384)rga-6(ok1316)* n = 100].

To investigate this compensatory mechanism further, we generated a series of double mutants for *lys-7* and the three candidate genes (*abl-1*, *fat-5* and *rga-6*) identified by resistance analysis, and tested their susceptibility to infection with *C. neoformans* and *S.* Typhimurium. Whilst *lys-7(ok1384);abl-1(ok171)* double mutants remained sensitive to killing by *C. neoformans*, the resistance to *S*. Typhimurium exhibited by both single mutants was completely abolished (Figs 3A and B). In contrast however, the other double mutants (*rga-6(ok1316);lys-7(ok1384)* and *fat-5(ok460);lys-7(ok1384)*) remained sensitive to killing by *C. neoformans* and resistant to killing by *S*. Typhimurium (Figs 3C and D), suggesting that the loss of *lys-7* is dominant in these animals, although we note that the degree of resistance has been slightly reduced in the case of *rga-6*. Taken together, these data indicate that *abl-1* and *lys-7* act together to regulate *S.* Typhimurium resistance in *C. elegans*.

### *S*. Typhimurium burden in the mutant animals is unchanged from wild type

**Figure 4:**
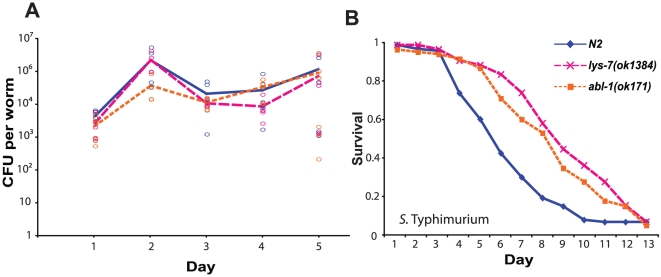
*lys-7* and *abl-1* mutant animals are tolerant of *S*. Typhimurium infection. (A) Bacterial load was assessed through viable counts. There was no difference between any of the strains, p>0.2, although there is a slight disparity between *abl-1(ok171)* and *lys-7(ok1384)* on day 4 of the infection, p<0.05, [*abl-1(ok171)* n = 60, *lys-7(ok1384)* n = 60; N2 n = 60]. (B) *lys-7(ok1384)* and *abl-1(ok171)* single mutants are tolerant to S. Typhimurium infection following a six-hour exposure, p<0.01 and p<0.0001 respectively, [*lys-7(ok1384)* n = 90; *abl-1(ok171)* n = 86; N2 n = 96].

We questioned whether the resistance of *lys-7(ok1384)* and *abl-1(ok171)* mutant animals to *S*. Typhimurium was due to a lower bacterial load within infected animals. To test this, we exposed wild type, *lys-7* and *abl-1* animals to *S*. Typhimurium L1019, a GFP-expressing derivative of SL1344 (kindly provided by Jessica Blair and Laura Piddock, University of Birmingham) and quantified infectious burden over time both through viable counts (Fig 4A) and microscopy (Fig S3). We found no consistent difference between any of the strains.

We also considered the possibility that *lys-7* and *abl-1* knockout animals may be better able to limit *S*. Typhimurium proliferation within the gut. To test this, we restricted the animals' exposure to the pathogen to just six hours and then shifted them to the normal feeding bacteria *E. coli* OP50 (a regime previously shown to lead to persistent colonisation [17]). Under these conditions both *lys-7* and *abl-1* animals retain their strong tolerance phenotypes (Fig 4B), but we observed no difference in *S.* Typhimurium colonisation between the mutants and wild type animals microscopically (Fig S4). Thus the enhanced survival of *lys-7* and *abl-1* knockout animals is due to an increased tolerance of *S*. Typhimurium within the gut, rather than resistance to infection or limitation of bacterial growth.

## Discussion

The lysozyme LYS-7 has been well-described in *C. elegans* as an essential antimicrobial molecule [7], [8], [9], [10]. Here we show that LYS-7 protects animals against *C. neoformans*-mediated killing, a function that is presumably attributable to the secondary chitinase (anti-fungal) activity exhibited by most lysozymes [19]. Remarkably, however, *lys-7* acts as a susceptibility factor for *S.* Typhimurium killing, as the loss of *lys-7* more than doubles the median survival of Salmonella-challenged animals.

In a manner similar to the *lys-7* mutant animals, we find that *abl-1* mutant worms are hypersusceptible to killing by *C. neoformans*. Since these animals show up-regulation of *lys-7*, a gene that we demonstrate to be important for cryptococcal resistance, we therefore conclude that ABL-1 regulates immunity at two levels (by the up-regulation of *lys-7* and by a second, *lys-7* independent pathway), both of which are required for wild type resistance to *C. neoformans*.

In vertebrates this phenomenon of immunological trade-off, whereby resistance to one class of pathogens comes at the cost of increased susceptibility to others, has been well documented [20], [21], [22]. Recently, analogous balance phenotypes have been described in *Drosophila* [23], [24], [25], [26], [27]. However, our finding of a susceptibility trade-off in *C. elegans*, mediated by the activity of LYS-7 and the tyrosine kinase ABL-1, is the first report of such a phenotype in nematodes and, as such, has significant implications for our understanding of the evolution of innate immunity in animals.

## Materials and Methods

### *C. elegans* and pathogen strains used

The strains used for this work are listed in Methods S1 and Table S1. Nematodes were cultured using standard methods as described previously [28], [29].

### *C. elegans* infection assays

20 µl of an overnight bacterial/fungal culture was inoculated onto 6 cm NGM plates, supplemented with the appropriate antibiotic if required, and lawns were allowed to grow at room temperature for 12 h. 15–30 animals at the L4 stage were picked onto each plate and subsequently transferred to newly seeded plates every 1–2 days. Survival was monitored every 24 hr and death was determined as a failure to respond to mechanical stimulus.

### *C. elegans* bacterial CFU analysis

Infection load was assessed through viable counts. The assay was modified from [30]; L4 animals were infected with *S*. Typhimurium strain L1019. At each timepoint, ten replicates of six animals each were incubated in 200 µl M9 buffer containing 25 mM levamisole hydrochloride (Sigma) and ampicillin (1 mg/ml) for one hour. The ampicillin was subsequently removed by three washes of 200 µl M9 with 25 mM levamisole hydrochloride. Animals were lysed in this buffer for 10 s using the Precellys 24 Lysis and Homogeniser. Lysates were serially diluted in M9 and plated onto LB plates containing kanamycin (30 µg/ml) to select for L1019. Colonies were counted by eye and scaled to the original concentration per nematode.

### *S*. Typhimurium persistence analysis

L4 animals were exposed to *S*. Typhimurium SL1344 on plates for six hours before being washed 3 times in M9 solution and shifted onto NGM plates seeded with 20 µl OP50 at 25°C. Animals were transferred to newly seeded plates every 1–2 days and scored for death (failure to respond to mechanical stimulus) every 1–2 days.

### RNA isolation, cDNA preparation and qRT-PCR

Total RNA was extracted from each strain on three independent occasions. Full plates of staged L4 animals were homogenised in 400 µl lysis buffer (Qiagen) using the Precellys 24 Lysis and Homogeniser. RNA was isolated from these lysates using the “RNeasy Mini Kit” (Qiagen) according to the manufacturer's instructions. RNA samples were treated with “DNA-*free*” (Ambion, Inc.) and subsequently quantified using the Nanodrop ND1000 microspectrophotometer (NanoDrop Technologies, Inc.). cDNA was synthesised using SuperScript II (Invitrogen) with random primers (Promega) in a SensoQuest Labcycler, assuming a 1∶1 conversion. The absence of genomic DNA was confirmed by PCR of these cDNA products (34 cycles: 94°C, 25 s; 55°C, 30 s; 72°C, 60 s), the amplimers of which were separated by electrophoresis on a 2.5% agarose gel. Quantitative real-time PCR (qRT-PCR) was performed on each cDNA sample in triplicate using 2x SensiMix (dU) SYBR Green kit (Quantace), but with a reaction volume of 25 µl. In each reaction MgCl_2_ had a final concentration of 3 mM, primers (sequences can be found in Table S2) were used at 20 mM and a template concentration of 2.5 ng was used. Each plate was run on an ABI Prism 7000 instrument with the following thermal cycling conditions: 37°C, 10 min; 95°C, 10 min; 95°C, 15 s; 55°C, 30 s; 72°C, 30 s; steps 3–5 were repeated for 40 cycles.

### Statistical analysis

All survival replicates were checked for consistency prior to being combined into single survival curves in Microsoft Excel using a macro-based template to calculate Kaplan-Meier survival probabilities every 24 hours. Each curve represents at least three independent experiments. Differences in survival were tested with a non-parametric log-rank analysis and assessed for significance using Chi squared. P-values below 0.05, after correcting for multiplicity, were taken to be significant. The data in the survival curves are presented as the proportion of animals surviving. The bacterial CFU analysis was carried out in Microsoft Excel and subsequently tested for significance using the Students' T-test (2 tailed, equal variance). The qRT-PCR was normalised to *gpd-3* expression using the Comparative Ct method in Microsoft Excel [31], [32], and tested for statistical significance using the Students' T-test (2 tailed, equal variance, paired).

## Supporting Information

Figure S1 ***lys-7*** **mutant nematodes do not differ from wild type animals in lifespan or brood size and the resistance phenotype is maintained with alternative Salmonella and** ***C. elegans*** **strains.** *lys-7* knockout animals have a normal lifespan in non-infectious conditions (A); p>0.2, [*lys-7(ok1384)* n = 129; N2 n = 199], and are unimpaired in their ability to produce young (B), data represent mean ± s.d. A further independent *lys-7* knockout strain (ok1385) showed the same balance phenotype, being hypersensitive to *C. neoformans* (C), p<0.001, [*lys-7(ok1385)* n = 101; N2 as Figure 1A] and resistant to *S*. Typhimurium SL1344 (D), p<0.0001, [*lys-7(ok1385)* n = 169; N2 as Figure 1D]. Moreover, the *lys-7(ok1384)* mutant worms exhibited the same enhanced resistance to infection with *S.* Typhimurium 14028 s (E) in comparison to the wild type strain; p<0.01, [*lys-7(ok1385)* n = 123; N2 n = 120].
(EPS)

Figure S2 ***abl-1*** **mutant nematodes do not show a reduction in lifespan or brood size and the resistance phenotype is maintained with alternative Salmonella and** ***C. elegans*** **strains.** Two further independent *abl-1* knockout strains showed the same balance phenotype, being hypersensitive to *C. neoformans* (A), p<0.0001 in both cases, [*abl-1(n1961)* n = 150; *abl-1(n1963)* n = 152; N2 n = 325] and resistant to *S*. Typhimurium SL1344 (B), p<0.0001 in both cases, [*abl-1(n1961)* n = 150; *abl-1(n1963)* n = 127; N2 n = 278]. *abl-1* knockout animals have a normal lifespan under non-infectious conditions (C); p>0.2, [*abl-1(ok171)* n = 142; N2 n = 159], and are unimpaired in their ability to produce young (D), data represent mean ± s.d. Further, no difference between *abl-1(ok171)* and wild type survival upon *S. aureus* infection was detected (E), p>0.2, [*abl-1(ok171)* n = 120; N2 as Figure 1B]. Moreover, the *abl-1* mutant worms exhibited the same enhanced resistance to infection with *S.* Typhimurium 14028 s (E) in comparison to the wild type strain; p<0.001, [*abl-1(ok171)* n = 90; N2 n = 112].
(EPS)

Figure S3 **Microscopic analysis of a constant** ***S.*** **Typhimurium infection in** ***C. elegans.*** Bacterial load of each *C. elegans* strain was assessed microscopically using a GFP-expressing *S*. Typhimurium, strain L1019, across a five day infection. No difference in infection load was identified between the strains.
(EPS)

Figure S4 **Microscopic analysis of a six hour** ***S.*** **Typhimurium infection in** ***C. elegans.*** Bacterial load of each *C. elegans* strain was assessed microscopically using a GFP-expressing *S*. Typhimurium, strain L1019, across a five day infection. No difference in infection load was identified between the strains.
(EPS)

Table S1 **Bacterial and fungal strains and their respective growth requirements.**
(DOC)

Table S2 **Primer Sequences.**
(DOC)

Methods S1 **Methodology for those data presented in supporting information.**
(DOC)
