# Supplementary material for: A Two-Gene Balance Regulates Salmonella Typhimurium Tolerance in the Nematode Caenorhabditis elegans
Source: PLoS One. 2011 Mar 2;6(3):e16839. doi: 10.1371/journal.pone.0016839 (PMC3047536; doi:10.1371/journal.pone.0016839)
Supplement: Table S1 — Bacterial and fungal strains and their respective growth requirements. (DOC) [file pone.0016839.s005.doc]

|  | **Strain** | **Strain details** | **Overnight Culture Medium** | ***C. elegans* media** | **Source/Reference** |
| --- | --- | --- | --- | --- | --- |
| ***Escherichia coli*** | OP50 | Uracil auxotroph | LB (1% tryptone, 1% NaCl, 0.5% yeast extract),  shaking at 37°C | NGM | (Brenner, 1974) |
| ***Cryptococcus neoformans*** | H99  (serotype A) | Clinical isolate from Hodgkin’s disease patient, USA | YPG (1% yeast extract, 1% peptone, 2% glucose) + kanamycin (30μg/ml), shaking at 25°C. | NGM  + Kan (30μg/ml) | Joe Heitman, Duke University  (Franzot et al., 1999) |
| ***Pseudomonas aeruginosa*** | PAO1 | Standard reference strain, wound isolate | LB, shaking at 37°C | NGM | Piddock Lab, University of Birmingham  (Stover et al., 2000) |
| ***Staphylococcus aureus*** | NCTC 8532  (ATCC 12600) | Standard type strain, clinical isolate from pleural fluid | LB, shaking at 37°C | NGM | Piddock Lab, University of Birmingham  (Piddock et al., 1999) |
| ***Salmonella* Typhimurium** | SL1344 | Standard reference strain, calf isolate, 1978 | LB, shaking at 37°C | NGM | Piddock Lab, University of Birmingham  (Wray and Sojka, 1978) |
| ***Salmonella* Typhimurium** | 14028s  (ATCC 14028) | Standard reference strain, isolated from chicken tissues | LB, shaking at 37°C | NGM | Piddock Lab, University of Birmingham  (Bailey et al., 2008) |
| ***Salmonella* Typhimurium** | L1019 | SL1344 + GFP*mut2* | LB + kanamycin (30μg/ml),  shaking at 37°C | NGM  + Kan (30μg/ml) | Blair and Piddock, Unpublished, University of Birmingham |
