# Supplementary material for: A Two-Gene Balance Regulates Salmonella Typhimurium Tolerance in the Nematode Caenorhabditis elegans
Source: PLoS One. 2011 Mar 2;6(3):e16839. doi: 10.1371/journal.pone.0016839 (PMC3047536; doi:10.1371/journal.pone.0016839)
Supplement: Table S2 — Primer Sequences. (DOC) [file pone.0016839.s006.doc]

| **Gene** | **PCR Type** |  | **Primer sequence (5’-3’)** | **Product size (bp)** | **Source** |
| --- | --- | --- | --- | --- | --- |
| ***lys-7*** | Single-worm | Forward | tccatcaaaattggcaacaa | WT: 2400  KO: 1600 | This paper |
|  |  | Reverse | cggcgaaataaattttggaa |  |  |
|  | qRT-PCR | Forward | GTCTCCAGAGCCAGACAATCC | 143 | Kindly provided by Claudia Boehnisch |
|  |  | Reverse | CCAGTGACTCCACCGCTGTA |  |  |
| ***abl-1*** | Single-worm | External Forward | TTTTGCTTTCAACTCGCCTT | WT: 3300  KO: 800 | *C. elegans* Knockout Consortium |
|  |  | External Reverse | ATATGCCTCCCTCCTTTGCT |  |  |
|  |  | Internal Forward | GTCTTCTGCTTTCGAATCGG |  |  |
|  |  | Internal Reverse | TTCATATATCCACCGGCCAT |  |  |
| ***fat-5*** | Single-worm | Forward | TTGCCTCCGGCAAACAGACT | WT: 1100  KO: 300 | (Brock et al., 2006) |
|  |  | Reverse | ATTCTCAGGCTTGAGCTCAG |  |  |
| ***clec-60*** | Single-worm | Forward | tctgccatccagactgcagt | WT: 1400  KO: 900 | WormBase |
|  |  | Reverse | cttcctataccgaccgtcga |  |  |
| ***rga-6*** | Single-worm | External Forward | actgattttgaggtggtggc | WT: 3200  KO: 2400 | WormBase |
|  |  | External Reverse | taaaaccgggaatggagttg |  |  |
|  |  | Internal Forward | gtctcgccacgacgaattat |  |  |
|  |  | Internal Reverse | aaatttcagttcgcattccg |  |  |
|  |  | External Reverse | tgtcgtgtcgagaccaggta |  |  |
|  |  | Internal Forward | agcagatcgattgttgttcaag |  |  |
|  |  | Internal Reverse | ttggtcccaaaaaccaaaaa |  |  |
| ***gpd-1*** | qRT-PCR | Forward | TGAAGGGAATTCTCGCTTACACC | 154 | (Amrit et al., 2010) |
|  |  | Reverse | GAGTATCCGAACTCGTTATCGTAC |  |  |
