# Supplementary material for: A Two-Gene Balance Regulates Salmonella Typhimurium Tolerance in the Nematode Caenorhabditis elegans
Source: PLoS One. 2011 Mar 2;6(3):e16839. doi: 10.1371/journal.pone.0016839 (PMC3047536; doi:10.1371/journal.pone.0016839)
Supplement: Methods S1 — Methodology for those data presented in supporting information. (DOC) [file pone.0016839.s007.doc]

*C. elegans* strains used

The strains used in this work were N2 (*C. elegans* wild type; Bristol isolate), RB1285 *lys-7(ok1834)*, RB1286 *lys-7(ok1385)*, XR1 *abl-1(ok171)*, RB1252 *rga-6(ok1316)*, BX107 *fat-5(tm420)*, kindly provided by the *C. elegans* Genetics Center and the *C. elegans* Knockout Consortium. *clec-60(tm2319)* was kindly provided by the National BioResource Project from the Mitani laboratory in Tokyo. Two further strains, *abl-1(n1691)* and *abl-1(n1963)* were provided by the Horvitz laboratory, in Boston, MA. Double mutant strains were generated during the study by crossing: FB0016 and FB0017 both *lys-7(ok1384)abl-1(ok171)*, FB0018 *lys-7(ok1384)rga-6(ok1316)* and FB0019 *lys-7(ok1384)fat-5(tm420)*.

Lifespan analysis

15-30 animals at L4 stage were picked from 9cm animal stock plates onto 6cm NGM plates seeded with 30μl OP50 and incubated at 20ºC. Animals were transferred to new seeded plates every one to two days during the fertile stage. The plates were scored for survival each day using a Leica M7.5 stereomicroscope and death was determined as a failure to respond to a mechanical stimulus. Data are representative of 100-200 animals, exact numbers are presented in the figure legends.

Brood size analysis

L4 hermaphrodites were singled onto 6cm NGM plates seeded with OP50; three plates of each strain. The animals were transferred to new plates one to two times daily during the fertile period. The progeny on the previous plates were allowed to grow up to around the L3 stage when they were counted using a Leica M7.5 stereomicroscope; the total brood size for each hermaphrodite was determined once all plates had been counted.

Statistical analysis

Lifespan replicates were checked for consistency prior to being combined into single survival curves in Microsoft Excel using a macro-based template to calculate Kaplan-Meier survival probabilities every 24 hours. The curves represent data from at least three individual experiments. Differences in survival were tested with a non-parametric log-rank analysis and assessed for significance using Chi squared. P-values below 0.05, after correcting for multiplicity, were taken to be significant. The data in the lifespan curves are presented as the proportion of animals surviving.

*C. elegans* bacterial load microscopy analysis

Animals at the L4 stage were infected with GFP-expressing *S*. Typhimurium strain L1019, as per the survival assays, either as a constant or persistent-shift infection. After a defined period, five replicates of six worms were transferred onto agarose pads on a microscope slide and paralyzed with an M9 solution containing 10mM Sodium azide. These slides were then imaged on a Nikon SMZ1000 stereomicrosope at 4x magnification, with a HR Plan Apo lens. An intensilight C-HGFI (Nikon) with a GFP filter set were used for fluorescence illumination. Digital images were captured with Nikon DS-Fi1 camera and NIS elements (v3.0) software. Images were processed in Adobe Photoshop. Those images presented are representative of the data set.
